# New species and records of *Chimarra* (Trichoptera, Philopotamidae) from Northeastern Brazil, and an updated key to subgenus Chimarra (Chimarrita)

**DOI:** 10.3897/zookeys.491.8553

**Published:** 2015-03-26

**Authors:** Albane Vilarino, Adolfo Ricardo Calor

**Affiliations:** 1 Universidade Federal da Bahia, Instituto de Biologia, Departamento de Zoologia, PPG Diversidade Animal, Laboratório de Entomologia Aquática - LEAq. Rua Barão de Jeremoabo, 147, campus Ondina, Ondina, CEP 40170-115, Salvador, Bahia, Brazil

**Keywords:** Biodiversity, caddisflies, *Curgia*, description, Neotropics, phylogenetic relationships, taxonomy

## Abstract

Two new species of Chimarra (Chimarrita) are described and illustrated, Chimarra (Chimarrita) mesodonta **sp. n.** and Chimarra (Chimarrita) anticheira **sp. n.** from the Chimarra (Chimarrita) rosalesi and Chimarra (Chimarrita) simpliciforma species groups, respectively. The morphological variation of Chimarra (Curgia) morio is also illustrated. Chimarra (Otarrha) odonta and Chimarra (Chimarrita) kontilos are reported to occur in the northeast region of Brazil for the first time. An updated key is provided for males and females of the all species in the subgenus *Chimarrita*.

## Introduction

Philopotamidae Stephens, 1829 is a cosmopolitan family with approximately 1,270 described species in 19 extant genera. The family is comprised of three subfamilies: Rossodinae Özdikmen & Darilmaz, 2008 (endemic to Madagascar, with 16 species); Philopotaminae Stephens, 1829 (present in all biogeographic regions, >400 species) and the most diverse subfamily, Chimarrinae Rambur, 1842 (cosmopolitan, *ca.* 800 species). Chimarrinae contains three genera: *Edidiehlia* Malicky, 1993 (Oriental, monospecific); *Chimarrhodella* Lestage, 1925 (Neotropical, 12 species); and one of the largest caddisfly genera, *Chimarra* Stephens 1829 (cosmopolitan, *ca.* 780 species) (Wahlberg et al. 2014). The genus *Chimarra* is characterized by foretibial spurs reduced to one (spur formula 1:4:4), and by an anal loop in the hindwing, in which the 2A vein is looped to join the 1A vein (Blahnik 1998). *Chimarra* is divided into four subgenera, the cosmopolitan *Chimarra* Stephens, 1829, and the three primarily Neotropical subgenera *Curgia* Walker, 1860, *Chimarrita* Blahnik, 1997, and *Otarrha* Blahnik, 2002. Wahlberg and Johanson’s (2014) analysis confirmed the monophyly of the genus *Chimarra* and hypothesized an early Cretaceous origin, approximately 138 million years ago. They suggested the genus first arose in the Neotropical region, with a subsequent radiation through the Oriental, Palaearctic and Australasian regions, secondarily to the Nearctic region, and with several independent colonization events in the Afrotropics.

The genus *Chimarra* currently comprises 247 Neotropical described species (45 in Brazil) and was revised by Flint (1998) and Blahnik (1997, 1998, 2002). Subsequent descriptions of new species were provided by Bueno-Soria et al. (2001), Santos and Nessimian (2009), and Blahnik and Holzenthal (2012). There are 102 Neotropical species recognized in the subgenus *Chimarra*, 93 in the subgenus *Curgia*, 32 in the subgenus *Otarrha*, and 18 in the subgenus *Chimarrita*, and 4 *incertae sedis* species. In Brazil, there are 45 recorded species in the genus, 27 of these in the subgenus *Curgia*, 2 in *Otarrha*, 3 in *Chimarra*, 12 in *Chimarrita*, and 1 *incertae sedis* species, *Chimarra usitatissima* Flint, 1971.

The subgenus *Chimarrita* was previously divided into 3 species groups by Blahnik (1997): Chimarra (Chimarrita) maldonadoi, Chimarra (Chimarrita) rosalesi and Chimarra (Chimarrita) simpliciforma groups. Recently Kjer et al. (2014) infered a phylogeny of the genus *Chimarra* using molecular data. Most of the subgeneric groups established by Blahnik based on morphological characters were recovered, but Kjer et al. (2014) did not support the placement of the *maldonadoi* Group in the subgenus *Chimarrita*, but rather considered the group *incertae sedis*, within the genus.

Males in the subgenus bear a partially to nearly separated tergum X, have very short knoblike and basally fused preanal appendages, the anteroventral margin of segment IX projected and tapering, usually to end acute apex, an elongate and narrow ventral process on segment IX, and a phallotheca with several short curved phallic spines or a single elongate spine almost always with slight helical twist. Females belonging to the subgenus *Chimarrita* can be easily diagnosed by the presence of an elongate tergum IX and segment VIII without anterolateral apodemes.

In this paper descriptions, diagnoses, and illustrations of two new species of Chimarra (Chimarrita) are provided, which fall within the *Chimarra rosalesi* and *Chimarra simpliciforma* groups. An updated key for males and females of the subgenus *Chimarrita* is also presented, and a description of the morphological variations of Chimarra (Curgia) morio. New records of Chimarra (Otarrha) odonta Blahnik, 2002, and Chimarra (Chimarrita) kontilos Blahnik, 1997, are reported for the first time from the northeast region of Brazil.

## Material and methods

Ultraviolet light alcohol pan traps, UV lights placed in front of a white bed sheet, and Malaise traps were used to collect adults (Calor and Mariano 2012, Blahnik and Holzenthal 2004). Specimens collected by Malaise and pan traps were preserved in 80% ethanol. Other specimens were collected using ethyl acetate kill jars and pinned. Genitalia were cleared in lactic acid following Blahnik et al. (2007) or in a heated solution of 10% KOH (Blahnik and Holzenthal 2004). Prepared genitalia were transferred to micro vials with glycerin and examined with optical microscopy at 40–400 × magnification. Structures were traced in pencil with the use of a camera lucida (drawing tube) mounted on a microscope. Digitally scanned pencil sketches were used as a template and rendered in Adobe Illustrator® CS5. Morphological terminology follows that established by Blahnik (1997).

The phylogenetic placement of the new species was compared with the previous phylogeny of the subgenus *Chimarrita* presented by Blahnik (1997) using the same characters with the addition of the following characters of the male genitalia:

Character 37: lobes of tergum X (shape); 0 = not roundly inflated apically (Fig. 2D), 1 = roundly inflated apically, club-shaped (Fig. 4D).

Character 38: ventral margin of tergum X; 0 = without a subapical small projection (Fig. 2C), 1 = with a subapical small projection (Fig. 4C).

Character 39: ventral apex of phallotica (shape); 0 = without an excision (Fig. 4E), 1 = with an excision (see Fig. 15E in Blahnik and Holzenthal 2012).

Character 40: inferior appendage (apex); 0 = not distinctly curved mesally (Fig. 4G), 1 = distinctly curved mesally (see Fig. 14C in Blahnik and Holzenthal 2012).

Character 41: inferior appendage (shape, lateral view); 0 = without a markedly undulate aspect (Fig. 4C), 1 = with a markedly undulated aspect, bent downward basally, upward at midlength, subapically rounded downward and apically upward (see Fig. 14A in Blahnik and Holzenthal 2012).

We also corrected an error in the coding of character 36 from Blahnik (1997) (female genitalia: sternum IX) of the species *Chimarra camura* that was modified from state “0” (not sclerously fused to segment VIII) to “2” (sclerously fused to segment VIII, broadly fused at base). As previously coded the state "0" for this species contradicted Blahnik’s (1997) description and illustrations. In addition, species or life stages were added to the matrix (male and female of *Chimarra curvipenis* and *Chimarra latiforceps* and the female of *Chimarra camella*). The matrix can be found in the Suppl. material 1.

The matrix was rebuilt using Nexus Data Editor (NDE), version 5.0 (Page 2001). The phylogenetic analysis was carried out in Tree analysis using New Technology (TNT), version 1.1 (Goloboff et al. 2008). For the TNT Maximum Parsimony analysis, an exhaustive search was used (implicit enumeration option). *Chimarrhodella ulmeri* was designed outgroup species. Characters 32, 33 and 36 were ordered, all other characters were unordered. All character were unweighted. Bootstrap analysis was implemented based on a thousand replicate samples.

Types and additional material are deposited in the Museu de Zoologia, Universidade de São Paulo, São Paulo State, Brazil (MZSP), Museu de Zoologia da Universidade Federal da Bahia, Bahia State, Brazil (UFBA, Collection of Aquatic Insects), and University of Minnesota Insect Collection, Minnesota, USA (UMSP), as indicated in the species descriptions. New records are indicated with bold type in the Distribution section.

## Results

**Figure 1.**
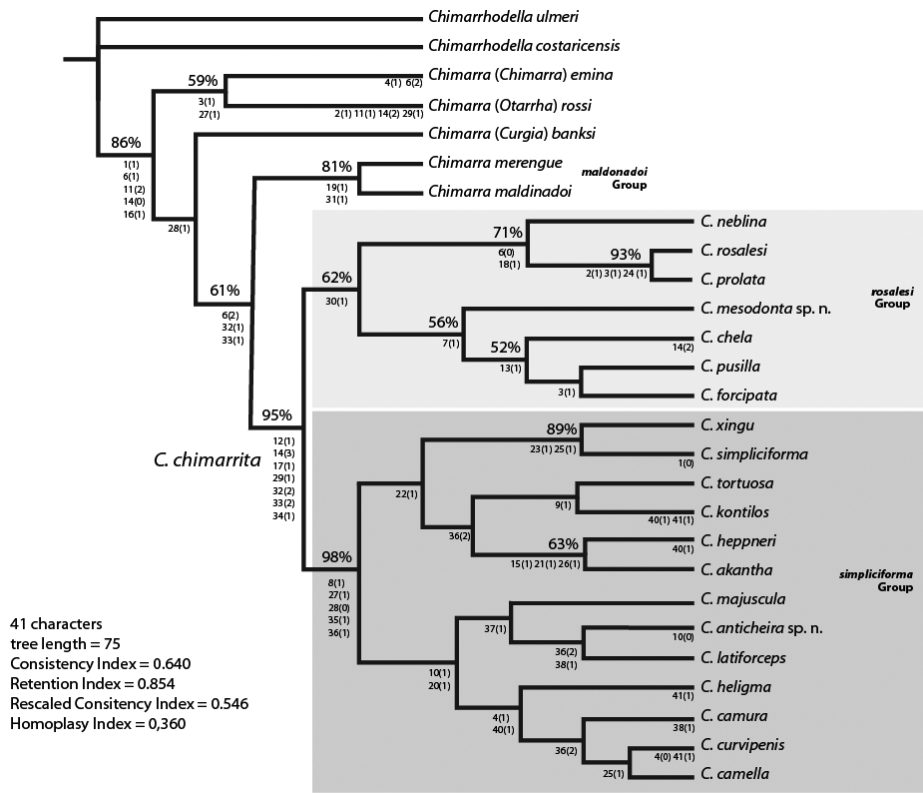
Cladogram for Chimarra (Chimarrita) species. Characters and character states are shown below and bootstrap support when >50% are shown above the base of clades.

The analysis resulted in a single parsimonious tree with a tree length of 75, consistency index of 0.640, rescaled consistency index of 0.854, retention index of 0.546, and homoplasy index of 0.360. The tree is presented in Fig. 1, with the synapomorphies and autapomorphies presented below the respective nodes. Bootstrap support values higher or equal to 50% are indicated above the respective nodes. The tree largely reflected the topology presented by Blahnik (1997) with additional characters solving the clade (*Chimarra majuscula* (*Chimarra anticheira*, *Chimarra latiforceps*) *Chimarra heligma*, (*Chimarra camura*, (*Chimarra curvipenis*, *Chimarra camella*))) but with low support (<50%). There was a reduction of the previous support value (61%) of the clade (*Chimarra tortuosa*, *Chimarra kontilos*) because of the similarities of the inferior appendage of *Chimarra kontilos*, *Chimarra heligma* and *Chimarra curvipenis* (character 41). Phylogenetic results concerning the new species are discussed under Remarks.

## Species descriptions

### Chimarra (Chimarrita) rosalesi Group

Synapomorphies recognized for this species group, both in the female genitalia are: segment VIII greatly reduced and membranous dorsally, and sternum IX not fused ventrally to segment VIII (Blahnik 1997). Similarities among males of the group are: anteroventral margin of segment IX elongate, usually tapering to acute apex, sometimes abruptly narrowed preapically; preanal appendages usually basally fused, sometimes ﬂattened and button-like; and phallic apparatus with spines (1 or more) usually short, curved and emerging more apically (Blahnik 1997). Currently, the *Chimarra rosalesi* Group comprises 7 species. The 6 previously described species are all distributed in the Amazon basin: *Chimarra chela*, *Chimarra neblina* (Venezuela and Amazonas states of Brazil), *Chimarra forcipata*, *Chimarra pusilla*, *Chimarra rosalesi* (Venezuela), and *Chimarra prolata* (Ecuador). *Chimarra mesodonta* sp. n. unlike the others was recorded from outlying fragment of the Atlantic Forest, Bahia State, Brazil.

#### Chimarra (Chimarrita) mesodonta

Taxon classificationAnimaliaTrichopteraPhilopotamidae

Vilarino & Calor sp. n.

http://zoobank.org/21106F6A-FBB4-4ED7-B8FF-2FCAFD41768F

Figs 2A–G , 3A–B

##### Diagnosis.

According to the phylogenetic analysis, *Chimarra mesodonta* has a sister relationship with the clade (*Chimarra chela* (*Chimarra pusilla*, *Chimarra forcipata*)) based on the presence of short phallic spines with a pronounced helical twist, but differs from these species by not sharing the flattened and button like preanal appendages. Among the species contained in this clade, this new species most closely resembles *Chimarra forcipata* by the overall aspect mainly in lateral view, and by both possess a mesally a mesally directed acute projection on the inferior appendage. *Chimarra mesodonta* can be distinguished from *Chimarra forcipata* by the following characters: R1 of the hind wing is not fused to the subcosta (fused in *Chimarra forcipata*); tergum X is shorter, and in lateral view slightly longer than the dorsal portion of segment IX (nearly 2× longer than segment IX in *Chimarra forcipata*); preanal appendage is not flattened (flattened in *Chimarra forcipata*); the ventral process is not strongly tapered, having apex subacute to truncate (strongly tapered and acute in *Chimarra forcipata*); in ventral view the apex of inferior appendage is broad and a projection is formed in the medial margin (apex strongly narrowed and with the projection formed apically in *Chimarra forcipata*); and the phallus bears 2 large helically curved spines (several small spines in *Chimarra forcipata*).

##### Description.

*Adult.* Forewing length 3.2–3.7 mm (males, n=5), 3.4–3.9 mm (females, n=6). Overall color (in alcohol) light brown. Forewing venation typical for *Chimarra*; Rs straight *s*, *r*, *r-m* and *m* of forewing unpigmented and linearly arranged, *m-cu* and apex of Cu2 also unpigmented; 2A apparently forked to 1A and 3A. Hind wing, R1 not fused to Sc; Rs 4-branched; M 3-branched. Posterior setal head warts large, triangular, meeting broadly on medial portion. Second segment of maxillary palp shorter than 3rd segment. Male pretarsal claws symmetrical, unmodified.

*Male genitalia.* Segment IX synsclerous; lateral view, anteroventral margin expanded, apex narrowed, acute; ventral process elongate, narrow, subacute, somewhat curved. Tergum X short, fused to segment IX; with short weakly sclerotized mesal lobe apically excavated, tergum X fully divided dorsally, separated ventrally until the length of mesal lobe, forming 2 separate, simple, lateral lobes with numerous sensilla. Preanal appendage very short, rounded, button-like, fused near base of tergum X. Inferior appendage short, triangular in lateral aspect; in ventral view, strongly rounded basally, tapered apically, apex rounded, medial margin forming tooth-like projection. Phallotheca tubular, slightly bent at middle, with rounded phallobase; with internal membranous structures and bearing 2 distinct curved phallic spines. Phallotremal sclerite complex could not be distinguished.

**Figure 2.**
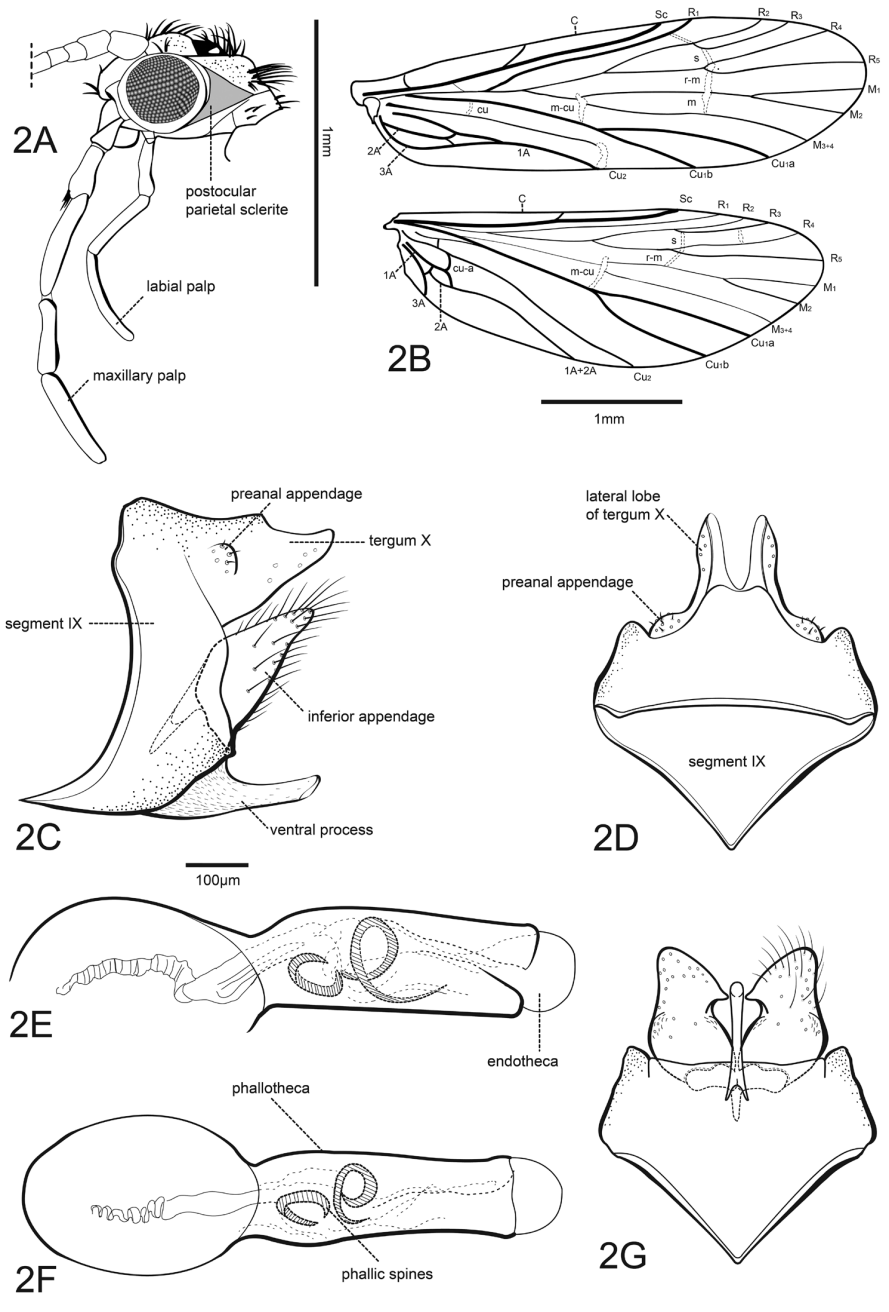
Chimarra (Chimarrita) mesodonta sp. n., male: **A** head, postocular parietal sclerite, maxillary and labial lateral **B** wing venation **C** genitalia, lateral **D** segment IX and tergum X, dorsal **E** phallic apparatus, lateral **F** dorsal **G** segment IX, inferior appendage, ventral.

*Female genitalia.* Sternum VII without ventral process. Segment VIII synsclerous, triangular in lateral aspect, dorsally membranous, very reduced; lateral suture line not evident, only demarcated by difference in texture and pigmentation of ventral portion, more granulous; anteroventral margin with subacute, deflected ventral process, posteroventral margin also with short ventral process. Sternum IX elongate, lightly sclerotized basally, with elongate, narrow, paired, ventral sclerites; sternum membranous between paired sclerites, and laterally from base to apex. Tergum IX elongate, narrow, nearly straight, sparsely setose, anteroventrally with short apodeme. Segment X with elongate basal portion, furrowed dorsally, with basal and inner margins more sclerotized, apically with small setose lobes, each with apical cercus. Vaginal apparatus largely membranous, anteriorly with weakly sclerotized structure.

**Figure 3.**
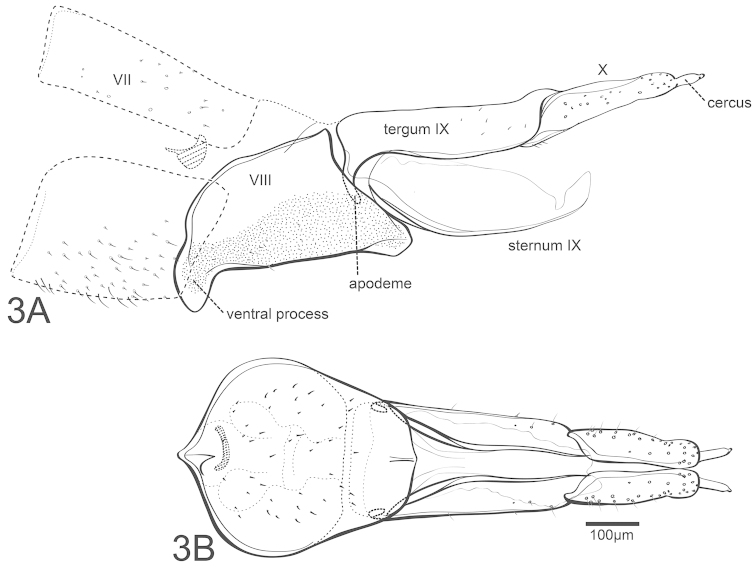
Chimarra (Chimarrita) mesodonta sp. n., female: **A** lateral **B** ventral.

##### Holotype, male

(alcohol)**. BRAZIL: Bahia:** Santa Teresinha, Pedra Branca, Serra da Jibóia, 12°51'016"S, 39°28'48"W, el. 679 m, 07.viii.2009, UV Light Pan trap, Calor A.R. & Lecci L.S. (MZUSP).

##### Paratypes.

**BRAZIL: Bahia:** same data as holotype, 2 males (alcohol) (UFBA); same, 12°51'00.6"S, 39°28'48.3"W, el. 678 m, 08.viii–28.ix.2009, Malaise trap, 1 female (alcohol) (UFBA); same, 04.ii.2010, Calor A.R. & Lecci L.S., 2 males, 2 females (alcohol) (UMSP); same, 10.vii.2010, UV Light Pan trap, Calor A.R. & Lecci L.S., 1 male (alcohol) (UFBA); same, 12°51'00.6"S, 39°28'48.3"W, el. 678 m, 19.vii.2009, Calor A.R. & Lecci L.S., 3 males (alcohol) (UFBA); Varzedo, Fazenda Vão da Serra, Riacho 2, 12°50'58.4"S, 39°28'04.0"W, el. 414 m, 08.ii.2014, UV Light Pan trap, Calor A.R. & Vilarino A., 16 males, 6 females (alcohol) (UFBA).

##### Etymology.

The species name is derived from the Greek *meso*, middle, and *donti*, tooth, referring to the median tooth present in the inferior appendages.

##### Remarks.

The aperture between the lateral lobes of tergum X can be wider depending on the specimen examined. Also, membranous structures of the endotheca and the lightly sclerotized mesal lobe of tergum X vary in shape depending on the preparation. When cleared in lactic acid, the basal portion of the endotheca shows some sclerotization and may be considered a rod of the phallotremal sclerite complex. Concerning the phylogenetic relationships, the new species shares character 30(1), segment VIII of female genitalia much narrowed or obsolete dorsally, with the *Chimarra rosalesi* Group *sensu* Blahnik (1997). The shared character 7(1), phallic spines short, with pronounced helical twist, supports the clade (*Chimarra mesodonta* (*Chimarra chela* (*Chimarra pusilla*, *Chimarra forcipata*))). However, the new species does not posses character 13(1), preanal appendages ﬂattened and button-like, a character shared by the other three species.

### Chimarra (Chimarrita) simpliciforma Group

Species belonging to this group are recognized by the presence of a single and elongate spine that emerges from the base of phallotheca. Females in the group have reduced or absent apodemes in tergum IX and sternum IX sclerously fused to segment VIII (Blahnik 1997). The *Chimarra simpliciforma* Group currently contains 12 species, with 11 previously described species distributed through the Amazon Region: *Chimarra akantha*, *Chimarra tortuosa* (Amazonas State of Brazil), *Chimarra simpliciforma* (Amazonas State of Brazil, Guyana, Surinam, and Venezuela), *Chimarra xingu* (Pará State of Brazil), *Chimarra heppneri* (Peru); and through Southeastern Brazil: *Chimarra camella* (Minas Gerais, Rio de Janeiro and São Paulo States) *Chimarra camura*, *Chimarra majuscula* (Rio de Janeiro and São Paulo states), *Chimarra curvipenis*, *Chimarra heligma* (Minas Gerais), *Chimarra kontilos* (Bahia, Espírito Santo, Minas Gerais, Rio de Janeiro and São Paulo states), *Chimarra latiforceps* (Minas Gerais and São Paulo states).

#### Chimarra (Chimarrita) anticheira

Taxon classificationAnimaliaTrichopteraPhilopotamidae

Vilarino & Calor sp. n.

http://zoobank.org/A1A073CB-3F97-430D-B851-A23AF4BCCCD2

Figs 4A–G , 5A–B

##### Diagnosis.

This species is very similar to *Chimarra latiforceps* Blahnik & Holzenthal, 2012, mainly by the general shape of tergum X that form apically clavate lobes. *Chimarra anticheira* can be distinguished from *Chimarra latiforceps* by the presence of a dorsally directed apical thumb-like projection on the inferior appendage (absent in *Chimarra latiforceps*). Additionally, the posterolateral margin of tergum IX is more angulate and the tergum X slightly shorter than in *Chimarra latiforceps*. The phallotheca in *Chimarra anticheira* is less curved than *Chimarra latiforceps* and the apicoventral portion of the phallotheca is rounded in *Chimarra anticheira*, whereas it is excavated in *Chimarra latiforceps*, also the apex of the phallic spine is angularly truncate (tapered in *Chimarra latiforceps*).

##### Description.

Forewing length 4.4–5.0 mm (males, n=5), 4.8–5.2 mm (females, n=5). Overall color (in alcohol) nearly uniformly medium brown. Forewing venation typical for *Chimarra*: Rs straight *s*, *r*, *r-m* and *m* of forewing unpigmented and linearly arranged, *m-cu* and apex of Cu2 also unpigmented; 2A apparently forked to 1A and 3A. Hind wing R1 not fused to Sc; Rs 4-branched, M 3-branched. Posterior setal head warts narrowly meeting on medial portion. Maxillary palps relatively short, 2nd segment longer than 3rd segment, 4th segment slightly bulbous. Male pretarsal claws symmetrical, unmodified.

*Male genitalia.* Tergum VIII short, forming sclerotized strip over segment IX. Segment IX synsclerous, anterodorsal margin excavate, rounded, posterolateral margin angularly projecting at level of inferior appendages, tapering to pointed apex; anteroventral margin expanded, with apex rounded in ventral aspect; ventral process elongate, narrow, acute, almost straight. Tergum X short, fused to segment IX, in dorsal view with apex mesally divided by rounded excision, extending about half of tergum length, forming paired lobes apically; apical lobes and lateral margins of tergum with many sensilla. Preanal appendage small, rounded, knob-like, positioned close to base of tergum X. Inferior appendage elongate, linear in lateral view, apex with thumb-like dorsally directed projection; in ventral view, appendages mesally curved, with submedial projection on mesal surface. Phallotheca tubular, with rounded phallobase; phallic spine single, elongate, emerging near phallobase, apparently fused with ventral portion of phallotheca, apex ventrally projecting, angularly truncate, pointed; endotheca forming sheath on basal half. Phallotremal sclerite complex appearing very narrow, elongate, apparently bifid sclerotized rod.

**Figure 4.**
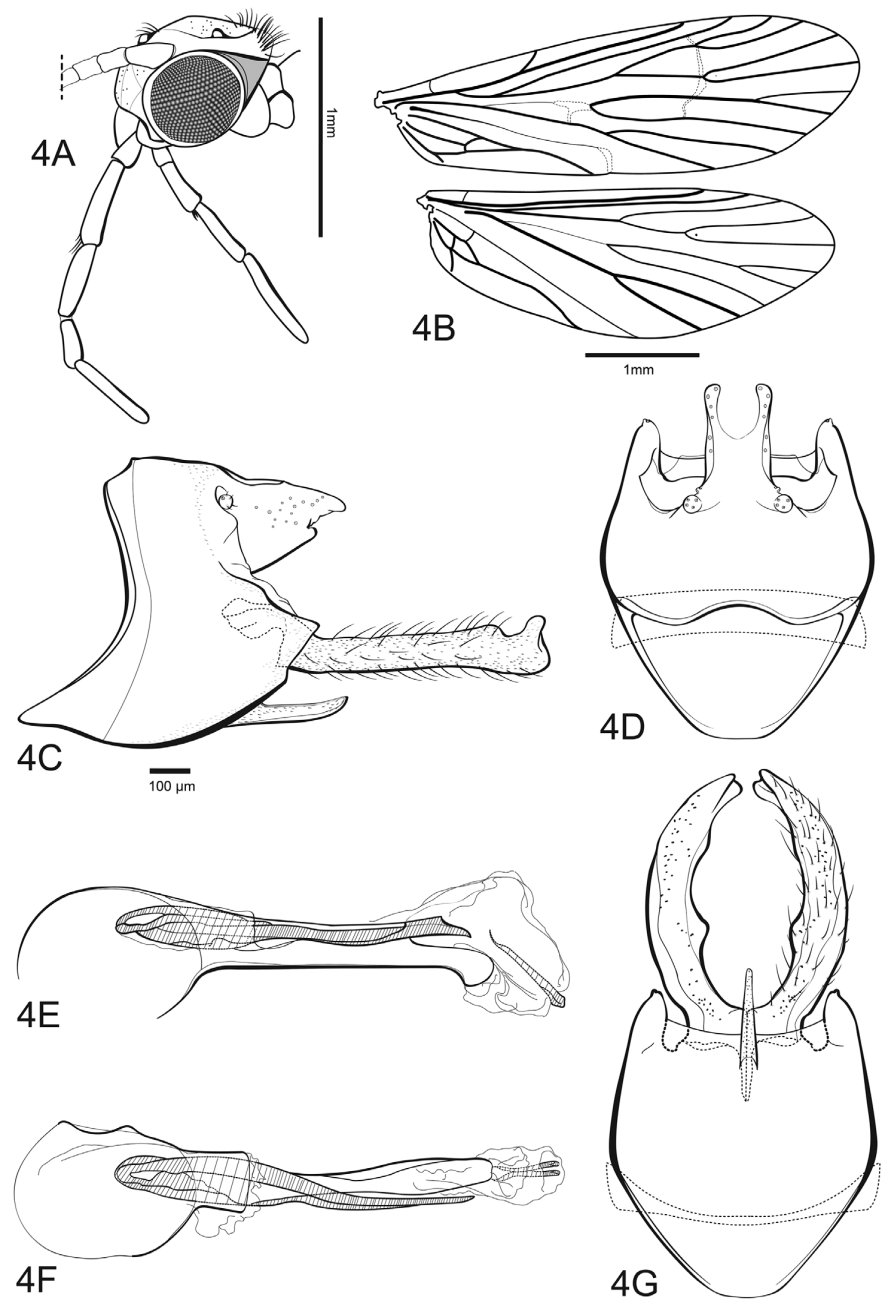
Chimarra (Chimarrita) anticheira sp. n., male: **A** head, postocular parietal sclerite, maxillary and labial lateral **B** wing venation **C** genitalia, lateral **D** segment IX and tergum X, dorsal **E** phallic apparatus, lateral **F** dorsal **G** segment IX, inferior appendage, ventral.

*Female genitalia.* Sternum VII with ventral process; process large, projecting, subacute, emerging close to middle of segment in lateral aspect. Segment VII synsclerous, short dorsally, anterolateral margin rounded, excavated dorsally and ventrally; segment fused ventrally to sternum IX; anteroventral margin of segment, as viewed ventrally, with short, narrow mesal emargination, margins of emargination distinctly sclerotized; segment bearing dorsal and ventral rounded unpigmented regions, usually around the larger setae. Sternum IX elongate, with paired, angular projections continuous posteriorly with elongate, narrow ventral sclerites; sternum membranous ventrally between the sclerites, and laterally from base until the apex. Tergum IX elongate, narrow, slightly curved, moderately setose, anteroventrally with short apodeme. Segment X with elongate basal portion, furrowed dorsally, with mesal tract of setae in furrow; apically with pair of small, rounded, setose lobes, each with short apical cercus. Vaginal apparatus largely membranous, with anterior sclerite forming ring and with pair of more elongate sclerites posteriorly.

**Figure 5.**
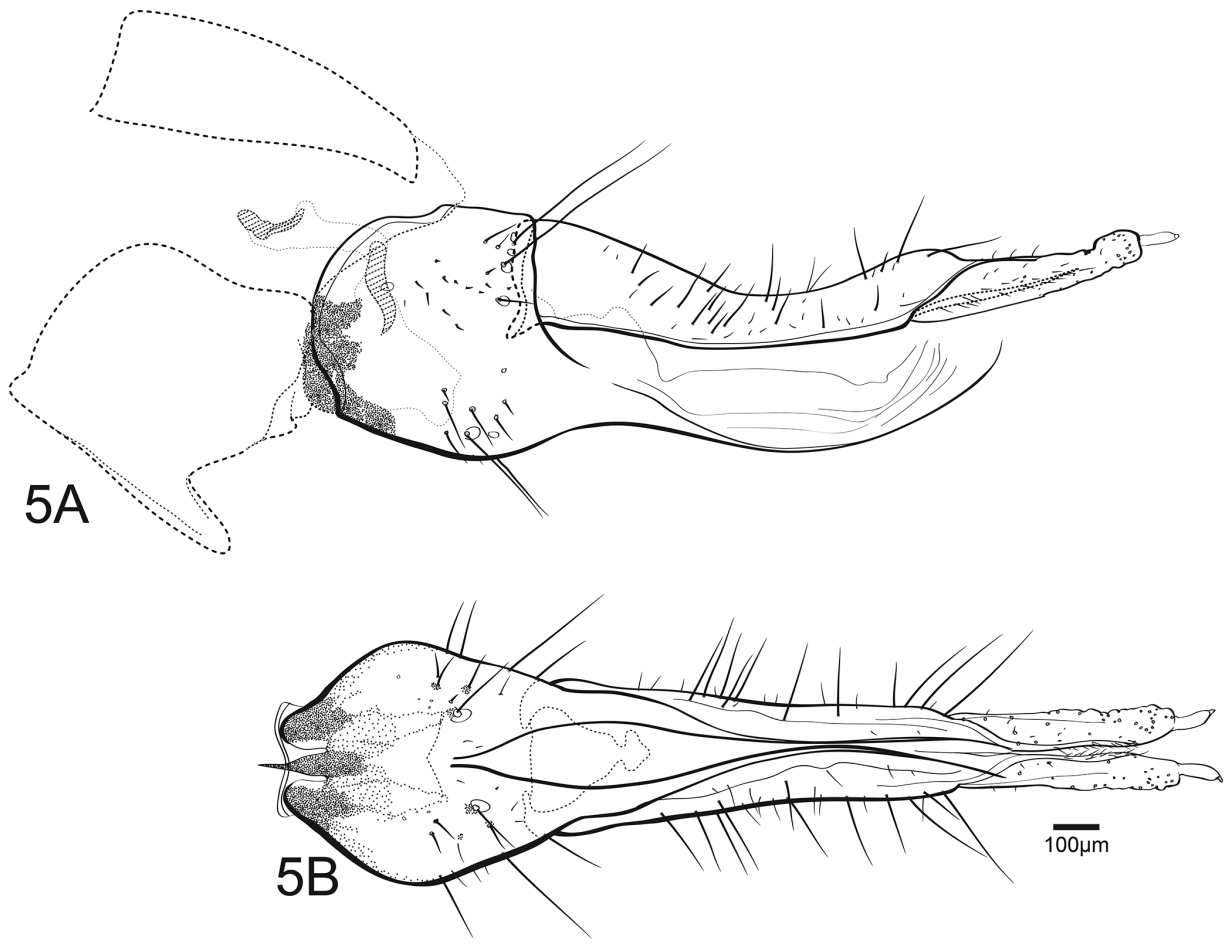
Chimarra (Chimarrita) anticheira sp. n., female: **A** lateral **B** ventral.

##### Holotype, male

(alcohol)**. BRAZIL: Bahia:** Varzedo, Fazenda Baixa Grande, Riacho Cai Camarão, Propriedade do Sr. Getúlio Rodrigues Leal, 12°57'45.1"S, 39°27'12.1"W, el. 280 m, 27.iii.2012, UV Light Pan trap, Quinteiro F.B., Duarte T., Garcia I. (MZUSP).

##### Paratypes.

**BRAZIL: Bahia:** same data as holotype, 2 males (alcohol) (UFBA); 12°57'39.2"S, 39°26'53.7"W, el. 252 m, 28.vi.2013, UV Light Pan trap, Calor A.R., Medeiros A. & Gomes V., 1 male (alcohol) (UFBA); 12°57'35.9"S, 39°26'54.9"W, el. 303 m, light, Calor A.R., Medeiros A. & Gomes V., 1 male, 3 females (alcohol) (UMSP); 12°57'35.9"S, 39°26'54.9"W, el. 303 m, 27.viii.2013, UV Light Pan trap, Calor A.R., Gomes V. & Zanata, A., 1 male (alcohol) (UFBA); 12°57'40.5"S, 39°26'54.7"W, el. 276 m, 09.ii.2014, UV Light Pan trap, Calor A.R. & Vilarino A., 3 males, 4 females (alcohol) (UFBA); 12°57'45.3"S, 39°27'13.1"W, el. 280 m, Calor A.R. & Vilarino A., 9 males (alcohol) (UFBA); Riacho Cachoeira da Serra (Monte Cruzeiro); 12°53'06.5"S, 39°26'47.1"W, el. 244 m, 08.ii.2014, Calor A.R. & Vilarino A., 1 male (alcohol) (UFBA); RPPN Guariru, Propriedade Flávio Pantaroto, 12°51'32.5"S, 39°27'59.5"W, el. 513 m, 07.ii.2014, UV Light Pan trap, Calor A.R. & Vilarino A., 9 females (alcohol) (UFBA); 12°51'33.1"S, 39°28'00.9"W, el. 524 m, Calor A.R. & Vilarino A., 17 males (alcohol) (UFBA); Cachoeira do Averaldo, 12°55'04.6"S, 39°26'39.7"W, el. 273 m, 09.ii.2014, Calor A.R. & Vilarino A., 2 males, 2 females (alcohol) (UFBA); Santa Teresinha, Pedra Branca, Serra da Jibóia, Riacho das torres, lajedo, 12°51'00.6"S, 39°28'48.3’W, el. 678 m, 10.vi.2010, UV Light Pan trap, Calor, A.R., 2 males, 2 females (alcohol) (UFBA); 28.ix.2009, light and sweep net, Calor A.R. & Cruz A.L. leg., 1 female (alcohol) (UFBA).

##### Etymology.

The species name derives from the Greek word *anticheira*, which means thumb, referring to the diagnostic thumb-like projection present on the inferior appendages.

##### Remarks.

Intra-specific variation may be observed in several structures. In some individuals the mesally formed projection on the inferior appendage is more prominent and there may also be a second subapical smaller projection. Membranous structures of the endotheca can vary in shape depending upon the preparation and some convoluted parts of phallic spines may be difficult to discern. The phylogenetic analysis resulted in a clade (*Chimarra majuscula* (*Chimarra anticheira*, *Chimarra latiforceps*)) based on the shared character 37(1), lobes of tergum X roundly inflated apicaly, club-shaped, and *Chimarra anticheira* as sister taxon of *Chimarra latiforceps* based on the characters 36(2), female genitalia with stemum IX sclerously fused to segment VIII, broadly fused at base, and 38(1), male genitalia with ventral margin of tergum X with a small projection subapically.

#### Chimarra (Curgia) morio

Taxon classificationAnimaliaTrichopteraPhilopotamidae

Burmeister, 1839

Figs 6A–F

Chimarrha morio Burmeister, 1839: 911 [Type locality: Brasilien; ZIUH, now lost; female; in *Chimarrha*]. — Ulmer 1905: 94. *Chimarra morio* (Burmeister). — Walker 1852: 8l [bibliography]. — Fischer 1961: 67 [bibliography]. — Flint 1998: 14 [male; redescription; variation; distribution; in *morio* group]. — Paprocki et al. 2004: 14 [checklist]. — Dumas et al. 2009: 313 [checklist]. — Calor 2011: 323 [checklist].
Chimarra martinmoselyi Botosaneanu, 1980: 98 [replacement name for *Chimarra moselyi* Ross, 1956, preoccupied by *Chimarra moselyi* Denning, 1947. Type locality: Argentina [sic, recte: Brazil], Petrópolis, Rio de Janeiro; BMNH; male]. — Flint 1998: 14 [to synonymy].

##### Observed intraspecific variation.

In his redescription, Flint (1998) analyzed specimens from diverse localities (Santa Catarina, Rio de Janeiro, São Paulo and also Bahia states) reporting wide variations, mainly in the shape and size of the inferior appendages. For the variant from Bahia, Flint (1998) the described inferior appendages and ventral process as longer, and tergum VII and tergum X as slightly bifid, but he did not provide any illustrations of this variant. Analysis of material from Bahia from the same locality reported by Flint and from other localities revealed other variations: Segment IX is opened dorsally forming a U-shape; in lateral aspect, the posterolateral margin of segment IX is slightly projected at level of inferior appendage. Tergum X in addition to being bifid is also constricted near the base, forming 2 small lateral lobes; the preanal appendages are elongated and tapered, with a small ventral projection; and the inferior appendages are rounded in ventral aspect, and have a medial and apical point on the lateral margin visible in lateral aspect.

##### Distribution.

Brazil (Bahia, Paraná, Rio de Janeiro, Santa Catarina, São Paulo).

##### Material examined.

**BRAZIL: Bahia:** Camacan, RPPN Serra Bonita, Riacho 1 trilha nova, 15°23'35.4"S, 39°33'50.1"W, el. 720 m, 01.iv.2011, UV Light Pan trap, Quinteiro F.B. & França D., 1 male (alcohol) (UFBA); same, 30.iii.2011, UV Light Pan trap, Quinteiro F.B. & França D., 1 male (alcohol) (UFBA); córrego 3, trilha 15°23'03"S, 39°34'00"W, el. 723 m, 29.x.2008, light, Calor A.R., Mariano R. & Mateus, 17 males, 9 females (alcohol) (UFBA); Elísio Medrado, Reserva Jequitibá, GAMBA, Córrego Caranguejo, 12°52'146"S, 39°28'337"W, el. 496 m, 08.xi.2010, Calor A.R., Quinteiro F.B., França D., Mariano R. & Costa A., 21 males (alcohol) (UFBA); Varzedo, Fazenda Baixa da Areia, Propriedade do Sr. Getúlio, Riacho Cai Camarão, 12°57'45.5"S, 39°26'55"W, el. 280 m, 27.iii.2012, Quinteiro F.B., Duarte T. & Garcia I., 44 males (alcohol) (UFBA); Riacho Cachoeira da Serra, 12°53'06.5"S, 39°26'47.0"W, el. 244 m, 08.ii.2014, UV Light Pan trap, Calor A.R. & Vilarino A., 2 males (alcohol) (UFBA); RPPN Guariru, 12°51'32.5"S, 39°27'59.5"W, el. 513 m, 07.ii.2014, UV Light Pan trap, Calor A.R. & Vilarino A., 2 males (alcohol) (UFBA); Santa Teresinha, Pedra Branca, córrego das torres, lajedo 12°51'016"S, 39°28'48"W, el. 679 m, 07.viii.2009, UV Light Pan trap, Calor A.R. & Lecci L.S., 2 males (alcohol) (UFBA).

**Figure 6.**
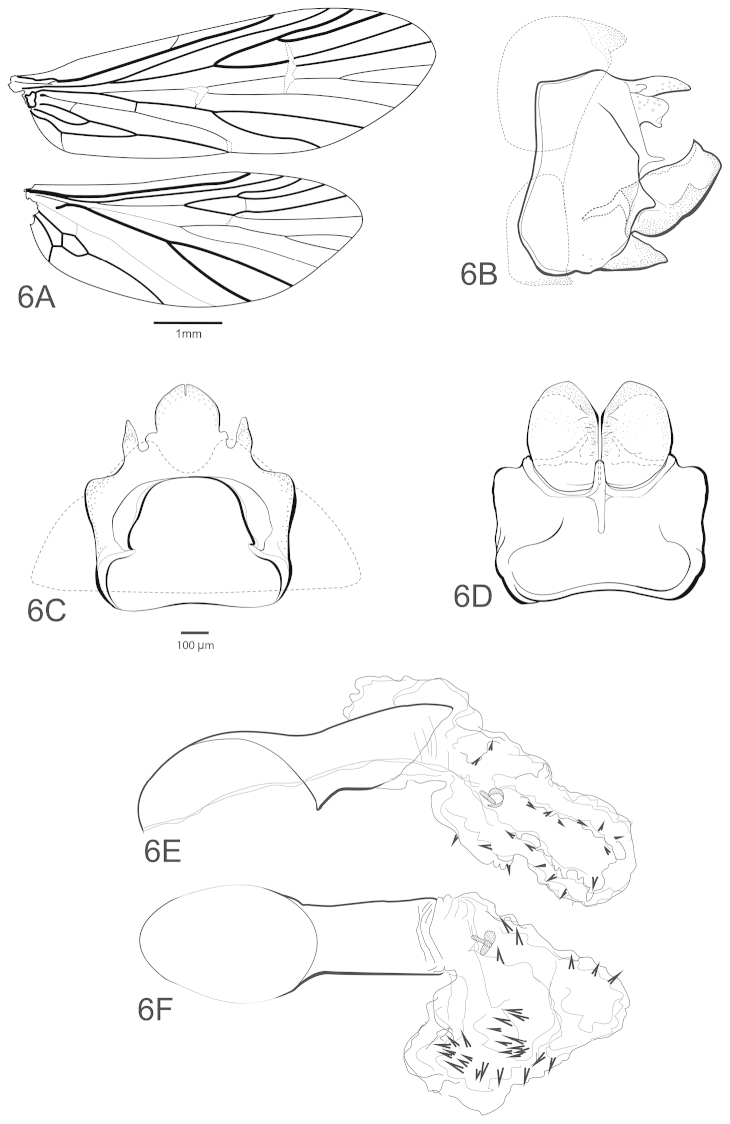
Chimarra (Curgia) morio. Male: **A** wing venation; male genitalia **B** lateral aspect **C** segment IX and tergum X, dorsal **D** segment IX, inferior appendage, ventral **E** phallic apparatus, lateral **F** dorsal.

### New records

#### Chimarra (Otarrha) odonta

Taxon classificationAnimaliaTrichopteraPhilopotamidae

Blahnik, 2002

Chimarra (Otarrha) odonta Blahnik, 2002: 85 [Type locality: Brazil, São Paulo, Est. Biol. Boracéia; MZUSP; male; female]. — Paprocki et al. 2004: 14 [checklist]. — Dumas et al. 2009: 364 [checklist]. — Calor 2011: 323 [checklist]. — Dumas et al. 2010: 8 [distribution]. — Barcelos-Silva et al. 2012: 1279 [distribution].

##### Distribution.

Brazil (Bahia [new record], Espírito Santo, Minas Gerais, Rio de Janeiro, São Paulo)

##### Material examined.

**BRAZIL: Bahia:** Camacan, RPPN Serra Bonita, riacho 1 trilha nova, 15°23'40"S, 39°33'44"W, el. 720 m, 31.iii.2011, UV Light Pan trap, Quinteiro F.B., França D. & Barreto H., 1 male (alcohol) (UFBA); Santa Teresinha, Pedra Branca, Riacho das Torres, 12°51'00"S, 39°28'48"W, el. 679 m, 28.ix.2009 – 04.iii 2010, Malaise trap, Calor A.R. & Dias E.S., 4 males (alcohol) (UFBA); Elísio Medrado, Reserva Jequitibá, GAMBA, Córrego Caranguejo, 12°52'146"S, 39°28'337"W, el. 496 m, 08.xi.2010, Calor A.R., Quinteiro F.B., França D., Mariano R. & Costa A., 3 males, 1 female (alcohol) (UFBA); same, 12°52'21.5"S 39°28'56.5"W, 07.xi.2010, Silva-Neto, Araujo, 1 male (alcohol) (UFBA); same, 12°52'040"S, 39°28'276"W, el. 232 m, 30.iii.2012, UV Light Pan trap, Quinteiro F.B., Duarte T. & Garcia I., 1 male (alcohol) (UFBA).

#### Chimarra (Chimarrita) kontilos

Taxon classificationAnimaliaTrichopteraPhilopotamidae

Blahnik, 1997

Chimarra (Chimarrita) kontilos Blahnik, 1997: 227 [Type locality: Brazil, Espírito Santo, Caixa d’Agua, Santa Teresa; MZUSP; male; female; in *simpliciforma* group]. — Blahnik et al. 2004: 5 [distribution]. — Paprocki et al. 2004: 14 [checklist]. — Dumas et al. 2009: 363 [checklist]. — Calor 2011: 323 [checklist].

##### Distribution.

Brazil (Bahia [new record], Espírito Santo, Minas Gerais, Rio de Janeiro, São Paulo).

##### Material examined.

**BRAZIL: Bahia:** Camacan, RPPN Serra Bonita, Riacho 1 trilha nova, 15°23'35.4"S, 39°33'50.1"W, el. 720 m, 01.iv.2011, UV Light Pan trap, Quinteiro F.B. & França D., 1 male (alcohol) (UFBA).

### Key for males and females of Chimarra (Chimarrita) (modified from Blahnik 1997)

Blahnik (1997) established the subgenus for 18 species (currently 16 species, with species of *Chimarra maldonadoi* Group moved to *incertae sedis*). Subsequently, two new species and the female of *Chimarra camella* were described by Blahnik and Holzenthal (2012). Here, two additional new species are described. An updated key of Chimarra (Chimarrita) is presented, now including all 20 known species (19 females).

**Table:** 

1	Inferior appendages (claspers) present; genitalia not elongate, attenuate (males)	**2**
–	Inferior appendages absent; genitalia elongate, attenuate (females)	**21**
2(1)	Phallic apparatus with spine(s) emergent from apex of phallotheca (see fig. 6E in Blahnik 1997) (elongate only in *Chimarra prolata*, see fig. 8E in Blahnik 1997) (*Chimarra rosalesi* Group)	**3**
–	Phallic apparatus with single spine, usually very elongate, emerging from base of phallotheca (see fig 18E in Blahnik 1997) (*Chimarra simpliciforma* Group)	**9**
3(2)	Phallic apparatus with spines not curved, or only slightly curved (see fig. 7E in Blahnik 1997)	**4**
–	Phallic apparatus with one or more helically curved, short spines (see fig. 5E in Blahnik 1997)	**6**
4(3)	Tergum X, in dorsal view, not elongate, cleft all the way to base; phallotheca short, slightly curved; inferior appendage elongate, narrow, apically acute (see fig. 7 in Blahnik 1997)	***Chimarra neblina* Blahnik**
–	Tergum X, in dorsal view, elongate narrow, cleft only in apical half; phallotheca elongate, tubular	**5**
5(4)	Inferior appendage elongate, tapering to acute apex; phallic apparatus with elongate spine, emerging apically; anteroventral margin of segment IX dramatically elongate, apex acute (see fig. 8 in Blahnik 1997)	***Chimarra prolata* Blahnik**
–	Inferior appendage, in dorsal view, with broadly rounded mesal projection at apex; phallic apparatus with short spines; ventral margin of segment IX elongate, acute, but not dramatically so (see fig. 10 in Blahnik 1997)	***Chimarra rosalesi* Flint**
6(5)	Inferior appendage, in lateral view, with deeply incised apex, producing curved, acute dorsal lobe, and shorter, acute ventral lobe (chelate, like lobster claw); anteroventral margin of segment IX with an obtuse apex (see fig. 5 in Blahnik 1997)	***Chimarra chela* Blahnik**
–	Inferior appendage without a incised apex, apex tapering (see fig. 6C in Blahnik 1997); anteroventral margin of segment IX with an acute apex (see fig. 6B in Blahnik 1997)	**7**
7(8)	Inferior appendage, in dorsal/ventral views, apically with rounded incurvature and acute apex; phallic apparatus with one curved spine (see fig. 9 in Blahnik 1997)	***Chimarra pusilla* Blahnik**
–	Inferior appendage, in dorsal/ventral views, with mesal acute projection; phallic apparatus with more than one curved spines (Fig. 2F, G)	**8**
8(9)	Inferior appendage, in dorsal/ventral views, strongly tapered apically, mesal projection emerging apically; phallic apparatus with several small curved spines; ventral process, in lateral view, very narrow and acute; preanal appendages flattened (see fig. 6 in Blahnik 1997)	***Chimarra forcipata* Blahnik**
–	Inferior appendage, in dorsal/ventral views, not strongly tapered, rounded apically, mesal projection emerging mesally about half appendage length; phallic apparatus with 2 large curved spines; ventral process, in lateral view, subacute and not strongly narrow; preanal appendages not flattened, slightly emergent (Fig. 2)	***Chimarra mesodonta* sp. n.**
9(2)	Phallotheca very elongate, narrow; phallic spine enormously elongate and sinuously curved, longer than phallotheca (see fig. 19E in Blahnik 1997)	**10**
–	Phallotheca only moderately elongate; phallic spine shorter and less sinuously curved, although often equaling length of phallotheca (see fig. 11E in Blahnik 1997)	**12**
10(9)	Inferior appendage with apex angularly incurved, apex not acute; apicoventrally with small, sclerous bidentate projection; phallotheca extremely elongate; phallic spine with retrorse, whip-like projection at apex (see fig. 16 in Blahnik 1997)	***Chimarra kontilos* Blahnik**
–	Inferior appendage with apex incurved and acute; phallotheca and phallic spine shorter; apex of phallic spine without retrorse, whip-like projection (see Fig. 14 in Blahnik and Holzenthal 2012)	**11**
11(10)	Tergum X with a small lateral, sensilla-bearing projection; inferior appendages strongly incurved apically; phallotheca strongly curved (see fig. 14 in Blahnik and Holzenthal 2012)	***Chimarra curvipenis* Blahnik & Holzenthal**
–	Tergum X without lateral sensilla-bearing projection; inferior appendages lightly incurved apically; phallotheca not strongly curved (see fig. 19 in Blahnik 1997)	***Chimarra tortuosa* Blahnik**
12(9)	Phallic spine short, much shorter than phallotheca; inferior appendage, in lateral view, abruptly and dramatically narrowed in apical half, apex dorsoventrally flattened; in dorsal/ventral views, with apex angularly incurved and rounded (see fig. 15 in Blahnik 1997)	***Chimarra heppneri* Blahnik**
–	Phallic spine elongate, nearly as long as phallotheca (see fig. 18E in Blahnik 1997); inferior appendage not as above	**13**
13(12)	Phallic apparatus with numerous, short, sclerous spines; inferior appendage, in lateral view, wide at apex and shallowly incised, forming subequal dorsal and ventral lobes; dorsal lobe with short, sclerous, mesally directed hook; ventral lobe with acute apex (see fig. 11 in Blahnik 1997)	***Chimarra akantha* Blahnik**
–	Phallic apparatus without short, sclerous spines (see fig. 18E in Blahnik 1997); inferior appendage not as above (if bilobed at apex, then without dorsal hook-like process)	**14**
14(13)	Phallotheca angularly flexed at base (see fig. 17E in Blahnik 1997); posterior margin of segment IX, in lateral view, very angularly protruding at level of inferior appendages (see fig. 17A in Blahnik 1997)	**15**
–	Phallotheca with slight curvature, but not angularly flexed at base; posterior margin of segment IX nearly linear, not (or only slightly) protruding (see fig. 18A in Blahnik 1997)	**20**
15(l4)	Apex of inferior appendage attenuate and curled inward; apex of lateral lobes of tergum X forming a spine-like lateral projection (see fig. 14D in Blahnik 1997)	***Chimarra heligma* Blahnik**
–	Apex of inferior appendage bluntly rounded, not attenuate; apex of lateral lobes of tergum X not as above (see fig. 13D in Blahnik 1997)	**16**
16(15)	Apex of inferior appendage distinctly cupped; tergum X with sensillate lateral protrusion (see figs 12B, D in Blahnik 1997)	***Chimarra camella* Blahnik**
–	Apex of inferior appendage flattened or angulate, not distinctly cupped; sensilla of tergum X not on rounded lateral protrusion (see figs 13B, D in Blahnik 1997)	**17**
17(l6)	Lateral lobes of tergum X with apex forming 2 points, inferior appendage only moderately elongate; in dorsal/ventral views, angularly incurved at apex, apex dorsoventrally flattened (see fig. 13A, B, D in Blahnik 1997)	***Chimarra camura* Blahnik**
–	Lateral lobes of tergum X with apex enlarged and rounded, club-shaped; inferior appendage distinctly elongate; in dorsal/ventral views, slightly incurved at apex, apex not dorsoventrally flattened (see fig. 17D in Blahnik 1997)	**18**
18(17)	Lateral lobes of tergum X very inflated apically, excision between the lobes about the same width as the apical portion of the lobes; apex of inferior appendage, flattened, apex without projection (see fig. 17B, D in Blahnik 1997)	***Chimarra majuscula* Blahnik**
–	Lateral lobes of tergum X narrow, slightly inflated apically, excision between the lobes about twice wider than the apical portion of the lobes; apex of inferior appendage broader and truncate (see fig. 15A, C in Blahnik and Holzenthal 2012) or with a dorsal thumb-like projection (Fig. 4)	**19**
19(18)	Phallotheca almost linear, apicoventral portion rounded; inferior appendages, in lateral view, with apicodorsal thumb-like projection (Fig. 4)	***Chimarra anticheira* sp. n.**
–	Phallotheca curved, bulbous, with apicoventral portion excavated; inferior appendage with truncate apex, without projection (see fig. 15E in Blahnik and Holzenthal 2012)	***Chimarra latiforceps* Blahnik & Holzenthal**
20(14)	Inferior appendage simple, short, apex rounded (see fig. 20D in Blahnik 1997)	***Chimarra xingu* Blahnik**
–	Inferior appendage with apex bifurcate, forming acute dorsal and ventral lobes (see fig. 18A in Blahnik 1997)	***Chimarra simpliciforma* Flint**
21(1)	Segment VIII with ventral process and very reduced or obsolete dorsally; segment VII lacking ventral process (see figs 9G, 10G in Blahnik 1997) (*Chimarra rosalesi* Group)	**22**
–	Segment VIII lacking ventral process and short, but not obsolete dorsally; segment VII with ventral process (see fig. 17G in Blahnik 1997) (*Chimarra simpliciforma* Group)	27*
22(21)	Tergum IX extremely elongate (8 or more times as long as high); ventral process short (see fig. 7G in Blahnik 1997)	**23**
–	Tergum IX elongate but not extremely so (approximately 6 times as long as high, or less); ventral process prominent (see fig. 5G in Blahnik 1997)	**24**
23(22)	Segment VIII, in lateral view, with anterior margin angular; segment subquadrate in shape (see fig. 10G in Blahnik 1997)	***Chimarra rosalesi* Flint**
–	Segment VIII, in lateral view, with anterior margin linear; segment triangular in shape (see fig. 7G in Blahnik 1997)	***Chimarra neblina* Blahnik**
24(22)	Segment VIII, in lateral view, with posterior margin nearly linear, not produced; ventral surface of segment not elongate; ventral process elongate, prominent, nearly as long as segment (see fig. 6G in Blahnik 1997)	***Chimarra forcipata* Blahnik**
–	Segment VIII, in lateral view, with posterior margin produced; ventral surface of segment elongate; ventral process not elongate, much shorter than segment (see fig. 9G in Blahnik 1997)	**25**
25(24)	Segment VIII, in lateral view, with posterior margin angularly produced, segment subquadrate in shape (see fig. 9G in Blahnik 1997)	***Chimarra pusilla* Blahnik**
–	Segment VIII, in lateral view, with posterior margin nearly linearly produced; subtriangular in shape (see fig. 5G in Blahnik 1997)	**26**
26(29)	Segment VIII, with two ventral processes an anterior and a small posterior one (Fig. 3)	***Chimarra mesodonta* sp. n.**
–	Segment VIII, with only one anterior ventral process (see fig. 5G in Blahnik 1997)	***Chimarra chela* Blahnik**
27(25)	Tergum IX moderately elongate, angularly downcurved from base (point of maximum flexion almost exactly in middle); sternum IX, in lateral view, with basodorsal angle posteriorly directed; sternum IX, in lateral view, narrow at point of articulation with segment VIII; ventral process of segment VII located posteriorly (see fig. 18G in Blahnik 1997)	**28**
–	Tergum IX moderately to distinctly elongate, less angularly downcurved (point of maximum flexion closer to apex than base, fig. 17G in Blahnik 1997); sternum IX, in lateral view, with basodorsal angle anteriorly or dorsally directed; sternum IX narrow or wide at point of articulation with segment VIII; ventral process of segment VII located either at posterior apex, or preapically (see fig. 15G in Blahnik 1997)	**29**
28(27)	Segment VIII with anterior margin more or less uniformly rounded (see fig. 20G in Blahnik 1997)	***Chimarra xingu* Blahnik**
–	Segment VIII with distinct bulge in anterior margin (see fig. 18G in Blahnik 1997)	***Chimarra simpliciforma* Flint**
29(27)	Ventral process of segment VII distinctly preapical (see fig. 17G in Blahnik 1997)	**30**
–	Ventral process of segment VII at or near posterior margin (see fig. 15G in Blahnik 1997)	**37**
30(29)	Segment IX with anteroventral margin moderately or weakly indentate, apodeme very small or somewhat developed, when anteroventral margin moderately indentate apodeme more developed and not pointed (see fig. 13G in Blahnik 1997)	**31**
–	Segment IX with anteroventral margin strongly indentate, with apodeme very narrow, small and pointed (see fig. 14G in Blahnik 1997)	**36**
31(30)	Basodorsal angle of sternum IX very angular and conspicuously sclerous; segment VIII, in lateral view, apparently narrowly connected to sternum IX (see fig. 17G in Blahnik 1997)	***Chimarra majuscula*** Blahnik
–	Basodorsal angle of sternum IX less distinct and segment IX more broadly connected to sternum IX (see fig. 13G in Blahnik 1997)	**32**
32(31)	Segment IX with anterior margin distinctly trapezoidal and extending about half length of the segment VIII (in lateral view); apodeme very reduced, almost obsolete (see fig 16G in Blahnik 1997)	***Chimarra kontilos* Blahnik**
–	Segment IX with anterior margin not distinctly trapezoidal and extending much less than half length of the segment VIII (in lateral view); apodeme developed (see fig 13G in Blahnik 1997)	**33**
33(32)	Segment IX with anterior margin smooth, without indentation, not extending over segment VIII (in lateral view); apodeme small and narrow (see fig. 13G in Blahnik 1997)	***Chimarra camura* Blahnik**
–	Segment IX with anterior margin weakly indentate or not indentate, extending slightly over segment VIII (in lateral view); apodeme a little broader (Fig. 5A)	**34**
34(33)	Segment VIII with anteroventral margin with rounded mesal emargination extending almost entire length of segment (in ventral view); anterolateral margin almost straight not elongate (in lateral view) (see fig. 16 in Blahnik and Holzenthal 2012)	***Chimarra curvipenis* Blahnik & Holzenthal**
–	Segment VIII with anteroventral margin with subquadrate mesal emargination, extending about half length of segment (in ventral view); anterolateral margin elongate rounded overall (in lateral view) (Fig. 5A)	**35**
35(34)	Ventral process of segment VII curved; apodeme longer, about half height of tergum IX (see fig. 17 in Blahnik and Holzenthal 2012)	***Chimarra latiforceps* Blahnik & Holzenthal**
–	Ventral process of segment VII not curved; apodeme smaller than half height of tergum IX (Fig. 4A)	***Chimarra anticheira* sp. n.**
36(30)	Segment VIII with conspicuous, sclerous lateral suture line, segment IX with anteroventral margin extending more than half way across segment VIII, apodeme small and narrow (see fig. 14G in Blahnik 1997)	***Chimarra heligma* Blahnik**
–	Segment VIII without lateral suture line, segment IX with anteroventral margin extending less than half way across segment VIII; apodeme strongly reduced (see fig. 18 in Blahnik and Holzenthal 2012)	***Chimarra camella* Blahnik**
37(29)	Ventral margin of segment VIII with distinct ventral bulge, extending angularly to posterior angle of sternum IX (see fig. 15G in Blahnik 1997)	***Chimarra heppneri* Blahnik**
–	Ventral margin of segment VIII straight or only slightly rounded, if rounded then not extending to posterior angle of stemum IX (see fig. 11G in Blahnik 1997)	**37**
38(37)	Segment VIII, in lateral view, with posterior margin extending nearly linearly from dorsal margin to posterior angle of stemum IX (see fig. 19G in Blahnik 1997); ventral margin of segment VIII straight, or nearly so; fusion of sternum IX with segment VIII not marked by conspicuous basal suture	***Chimarra tortuosa* Blahnik**
–	Segment VIII, in lateral view, with posterior margin less distinctly linear; ventral margin of segment VIII slightly rounded; fusion of stemum IX with segment VIII marked by conspicuous basal suture (see fig. 11G in Blahnik 1997)	***Chimarra akantha* Blahnik**

* These species are all similar, and the characters used to separate them are presumptive based on the material available (Blahnik 1997).

## Supplementary Material

XML Treatment for Chimarra (Chimarrita) mesodonta

XML Treatment for Chimarra (Chimarrita) anticheira

XML Treatment for Chimarra (Curgia) morio

XML Treatment for Chimarra (Otarrha) odonta

XML Treatment for Chimarra (Chimarrita) kontilos

## Supplementary materials

Supplementary material 1 Matrix data table used in the cladistic analyses of Chimarra (Chimarrita). Data type: matrix data Explanation note: Matrix with 27 taxa and 41 characters used in the cladistic analyses of Chimarra (Chimarrita). Missing data are coded as “?”, characters 32, 33, 36 are ordered, and all other characters were unordered. This dataset is made available under the Open Database License (http://opendatacommons.org/licenses/odbl/1.0/). The Open Database License (ODbL) is a license agreement intended to allow users to freely share, modify, and use this Dataset while maintaining this same freedom for others, provided that the original source and author(s) are credited.Albane Vilarino, Adolfo Ricardo Calor

## References

[B1] Barcelos-Silva P Pes AMO Salles FF (2012) Annulipalpia (Insecta: Trichoptera) from the state of Espírito Santo, Brazil. CheckList 8: 1274–1279.

[B2] Blahnik RJ (1997) Systematics of *Chimarrita*, a new subgenus of *Chimarra* (Trichoptera: Philopotamidae). Systematic Entomology 22: 199–243. doi: 10.1046/j.1365-3113.1997.d01-39.x

[B3] Blahnik RJ (1998) A revision of the Neotropical species of the genus *Chimarra*, subgenus *Chimarra* (Trichoptera: Philopotamidae). Memoirs of the American Entomological Institute 59: vi+1–318.

[B4] Blahnik RJ (2002) Systematics of *Otarrha*, a new Neotropical subgenus of *Chimarra* (Trichoptera: Philopotamidae). Systematic Entomology 27: 65–130. doi: 10.1046/j.0307-6970.2001.00166.x

[B5] Blahnik RJ Holzenthal RW (2004) Collection and curation of Trichoptera, with an emphasis on pinned material. Nectopsyche, Neotropical Trichoptera Newsletter 1: 8–20.

[B6] Blahnik RJ Holzenthal RW (2012) New Neotropical species of *Chimarra* (Trichoptera, Philopotamidae). ZooKeys 184: 1–33. doi: 10.3897/zookeys.184.2911 22573949 10.3897/zookeys.184.2911 PMC3332012

[B7] Blahnik RJ Holzenthal RW Prather AL (2007) The lactic acid method for clearing Trichoptera genitalia. In: Bueno-Soria J Barba-Álvarez R Armitage BJ (Eds) Proceedings of the 12th International Symposium on Trichoptera. The Caddis Press, Columbus, Ohio, 9–14.

[B8] Blahnik RJ Paprocki H Holzenthal RW (2004) New distribution and species records of Trichoptera from Southern and Southeastern Brazil. Biota Neotropica 4: 1–6. doi: 10.1590/S1676-06032004000100009

[B9] Botosaneanu L (1980) Trichòpteres adultes de Cuba collectés par les zoologistes cubains (Trichoptera). Mitteilungen der Munchner Entomologischen Gesellschaft 69: 91–116.

[B10] Bueno-Soria J Santiago-Fragoso S Barba-Alvarez R (2001) Studies in aquatic insects, XVIII: new species and new record of caddisflies (Trichoptera) from Mexico. Entomological News 112: 145–158.

[B11] Burmeister H (1839) Trichoptera (Handbuch der Entomologie). Handbuch der Entomologie: 882–935.

[B12] Calor AR (2011) Checklist dos Trichoptera (Insecta) do Estado de São Paulo, Brasil. Biota Neotropica 11: 619–630. doi: 10.1590/S1676-06032011000500028

[B13] Calor AR Mariano R (2012) UV light pan traps for collecting aquatic insects. Entomobrasilis 5: 164–166. doi: 10.12741/ebrasilis.v5i2.187

[B14] Denning DG (1947) New Species of Trichoptera from the United States. Entomological News 58: 249–257.

[B15] Dumas LL Jardim GA Santos APM Nessimian JL (2009) Tricópteros (Insecta: Trichoptera) do Estado do Rio de Janeiro: lista de espécies e novos registros. Arq. Mus. Nac., Rio de Janeiro 67: 355–376.

[B16] Dumas LL Santos APM Jardim GA Ferreira N Jr Nessimian JL (2010) Insecta, Trichoptera: New records from Brazil and other distributional notes. CheckList 6: 7–9.

[B17] Fischer FCJ (1961) Philopotamidae, Hydroptilidae, Stenopsychidae. Trichopterum Catalogus II. Nederlandsche Entomologische Vereeniging, Amsterdam, iv+169 pp.

[B18] Flint OS Jr. (1964) The Caddisflies (Trichoptera) of Puerto Rico. Technical Paper 40: 6–77.

[B19] Flint OS Jr. (1971) Studies of Neotropical caddisflies. XII: Rhyacophilidae, Glossosomatidae, Philopotamidae, and Psychomyiidae from the Amazon Basin (Trichoptera). Amazonia 3(1): 1–67.

[B20] Flint OS Jr. (1998) Studies of Neotropical caddisflies, LIII: a taxonomic revision of the subgenus *Curgia* of the genus *Chimarra* (Trichoptera: Philopotamidae). Smithsonian Contributions to Zoology 594: 1–131. doi: 10.5479/si.00810282.594

[B21] Goloboff PA Farris JS Nixon KC (2008) TNT, a free program for phylogenetic analysis. Cladistics 24: 774–786. doi: 10.1111/j.1096-0031.2008.00217.x

[B22] Kjer KM Zhou X Frandsen PB Thomas JA Blahnik RJ (2014) Moving toward species-level phylogeny using ribosomal DNA and COI barcodes: an example from the diverse caddisfly genus Chimarra (Trichoptera: Philopotamidae). Arthropod Systematics & Phylogeny 72(3): 345–354.

[B23] Lestage JA (1925) Notes Trichoptérologiques (7me NOTE). Bulletin et Annales de la Société Entomologiques de Belgique 65: 35–44.

[B24] Malicky H (1993) Neue asiatische Kocherfliegen (Trichoptera: Philopotamidae. Polycentropididae, Psychomyidae, Ecnomidae, Hydropsychidae, Leptoceridae). Linzer biologischen Beiträgen 25: 1099–1136.

[B25] Özdikmen H Darilmaz MC (2008) New subfamily and genus names, Rossodinae nom. nov. and Rossodes nom. nov., for the finger-net caddisflies (Trichoptera: Philopotamidae). Munis Entomology & Zoology 3: 162–164.

[B26] Page RDM (2001) NDE (NEXUS data editor for windows). Version 0.5.0 NDE. http://taxonomy.zoology.gla.ac.uk/rod/NDE/nde.html [access in July 2014]

[B27] Paprocki H Holzenthal RW Blahnik RJ (2004) Checklist of the Trichoptera (Insecta) of Brazil I. Biota Neotropica 4: 1–22. doi: 10.1590/S1676-06032004000100008

[B28] Rambur JP (1842) Néuropteres. In: Histoire Naturelle des Insectes, Vol. 1 Libraire Encyclopedique de Roret [private printing], Paris, 534 pp.

[B29] Ross H (1956) Evolution and Classification of the Mountain Caddisflies. University of Illinois Press, Urbana, 213 pp.

[B30] Santos APM Nessimian JL (2009) New species and records of *Chimarra* Stephens (Trichoptera, Philopotamidae) from Central Amazonia, Brazil. Revista Brasileira de Entomologia 53: 23–25. doi: 10.1590/S0085-56262009000100006

[B31] Stephens JF (1829) A Systematic Catalogue of British Insects, Part I. Baldwin & Cradock, London, 416 pp.

[B32] Ulmer G (1905) Zur Kenntniss aussereuropäischer Trichopteren. Stettiner Entomologische Zeitung 66: 1–119.

[B33] Wahlberg E Espeland M Johanson KA (2014) Seven new species of *Chimarra* (Trichoptera: Philopotamidae) from Malawi. Zootaxa 3796: 579–93. doi: 10.11646/zootaxa.3796.3.10 24870694 10.11646/zootaxa.3796.3.10

[B34] Wahlberg E Johanson KA (2014) The age, ancestral distribution and radiation of *Chimarra* (Trichoptera: Philopotamidae) using molecular methods. Molecular Phylogenetics and Evolution 79: 433–442. doi: 10.1016/j.ympev.2014.06.023 25008108 10.1016/j.ympev.2014.06.023

[B35] Walker F (1852) Catalogue of the Specimens of Neuropterous Insects in the Collection of the British Museum, Part I: Phryganides-Perlides. British Museum, London, 192 pp.

[B36] Walker F (1860) XIII. Characters of undescribed Neuroptera in the collection of W. W. Saunders Esq. F. R. S. & C. Transactions of the Entomological Society of London 5: 176–199.

