# Supplementary material for: New species and records of Chimarra (Trichoptera, Philopotamidae) from Northeastern Brazil, and an updated key to subgenus Chimarra (Chimarrita)
Source: Zookeys. 2015 Mar 26;(491):119–42. doi: 10.3897/zookeys.491.8553 (PMC4389187; doi:10.3897/zookeys.491.8553)
Supplement: Supplementary material 1 — Matrix data table used in the cladistic analyses of Chimarra (Chimarrita). [file zookeys-491-119-s001.docx]

| Taxa | 1 | 2 | 3 | 4 | 5 | 6 | 7 | 8 | 9 | 10 | 11 | 12 | 13 | 14 | 15 | 16 | 17 | 18 | 19 | 20 |
| --- | --- | --- | --- | --- | --- | --- | --- | --- | --- | --- | --- | --- | --- | --- | --- | --- | --- | --- | --- | --- |
| *C. akantha* | 1 | 0 | 0 | 0 | 1 | 2 | 0 | 1 | 0 | 0 | 2 | 1 | 0 | 3 | 1 | 1 | 1 | 0 | 0 | 0 |
| *C. anticheira* sp. n. | 1 | 0 | 0 | 0 | 0 | 2 | 0 | 1 | 1 | 1 | 2 | 1 | 0 | 3 | 0 | 1 | 1 | 0 | 0 | 1 |
| *C. camella* | 1 | 0 | 0 | 1 | 0 | 2 | 0 | 1 | 0 | 1 | 2 | 1 | 0 | 3 | 0 | 1 | 1 | 0 | 0 | 1 |
| *C. camura* | 1 | 0 | 0 | 1 | 0 | 2 | 0 | 1 | 0 | 1 | 2 | 1 | 0 | 3 | 0 | 1 | 1 | 0 | 0 | 1 |
| *C. chela* | 1 | 0 | 0 | 0 | 0 | 2 | 1 | 0 | 0 | 0 | 2 | 1 | 1 | 2 | 0 | 1 | 1 | 0 | 0 | 0 |
| *C. curvipenis* | 1 | 0 | 0 | 0 | 0 | 2 | 0 | 1 | 0 | 1 | 2 | 1 | 0 | 3 | 0 | 1 | 1 | 0 | 0 | 1 |
| *C. forcipata* | 1 | 0 | 1 | 0 | 0 | 2 | 1 | 0 | 0 | 0 | 2 | 1 | 1 | 3 | 0 | 1 | 1 | 0 | 0 | 0 |
| *C. heligma* | 1 | 0 | 0 | 1 | 0 | 2 | 0 | 1 | 0 | 1 | 2 | 1 | 0 | 3 | 0 | 1 | 1 | 0 | 0 | 1 |
| *C. heppneri* | 1 | 0 | 0 | 0 | 1 | 2 | 0 | 1 | 0 | 0 | 2 | 1 | 0 | 3 | 1 | 1 | 1 | 0 | 0 | 0 |
| *C.kontilos* | 1 | 0 | 0 | 0 | 1 | 2 | 0 | 1 | 1 | 0 | 2 | 1 | 0 | 3 | 0 | 1 | 1 | 0 | 0 | 0 |
| *C. latiforceps* | 1 | 0 | 0 | 0 | 0 | 2 | 0 | 1 | 0 | 1 | 2 | 1 | 0 | 3 | 0 | 1 | 1 | 0 | 0 | 1 |
| *C. majuscula* | 1 | 0 | 0 | 1 | 0 | 2 | 0 | 1 | 0 | 1 | 2 | 1 | 0 | 3 | 0 | 1 | 1 | 0 | 0 | 1 |
| *C. maldonadoi* | 1 | 0 | 0 | 0 | 0 | 2 | 0 | 0 | 0 | 0 | 2 | 0 | 0 | 0 | 0 | 1 | 0 | 0 | 1 | 0 |
| *C. merengue* | 1 | 0 | 0 | 0 | 0 | 2 | 0 | 0 | 0 | 0 | 2 | 0 | 0 | 0 | 0 | 1 | 0 | 0 | 1 | 0 |
| *C. mesodonta* sp. n. | 1 | 0 | 0 | 0 | 0 | 2 | 1 | 0 | 0 | 0 | 2 | 1 | 0 | 3 | 0 | 1 | 1 | 0 | 0 | 0 |
| *C. neblina* | 1 | 0 | 0 | 0 | 0 | 0 | 0 | 0 | 0 | 0 | 2 | 1 | 0 | 3 | 0 | 1 | 1 | 1 | 0 | 0 |
| *C. prolata* | 1 | 1 | 1 | 0 | 0 | 0 | 0 | 0 | 0 | 0 | 2 | 1 | 0 | 3 | 0 | 1 | 1 | 1 | 0 | 0 |
| *C. pusilla* | 1 | 0 | 1 | 0 | 0 | 2 | 0 | 0 | 0 | 0 | 2 | 1 | 1 | 3 | 0 | 1 | 1 | 0 | 0 | 0 |
| *C. rosalesi* | 1 | 1 | 1 | 0 | 0 | 0 | 0 | 0 | 0 | 0 | 2 | 1 | 0 | 3 | 0 | 1 | 1 | 1 | 0 | 0 |
| *C. simpliciforma* | 0 | 0 | 0 | 0 | 1 | 2 | 0 | 1 | 0 | 0 | 2 | 1 | 0 | 3 | 0 | 1 | 1 | 0 | 0 | 0 |
| *C. tortuosa* | 1 | 0 | 0 | 0 | 1 | 2 | 0 | 1 | 1 | 0 | 2 | 1 | 0 | 3 | 0 | 1 | 1 | 0 | 0 | 0 |
| *C. xingu* | 1 | 0 | 0 | 0 | 1 | 2 | 0 | 1 | 0 | 0 | 2 | 1 | 0 | 3 | 0 | 1 | 1 | 0 | 0 | 0 |
| *Chimarrhodella ulmeri* | 0 | 0 | 0 | 0 | 0 | 0 | 0 | 0 | 0 | 0 | 0 | 0 | 0 | 1 | 0 | 0 | 0 | 0 | 0 | 0 |
| *Chimarrhodella costaricensis* | 0 | 0 | 0 | 0 | 0 | 0 | 0 | 0 | 0 | 0 | 0 | 0 | 0 | 1 | 0 | 0 | 0 | 0 | 0 | 0 |
| *Chimarra* (*Curgia*) *banksi* | 1 | 0 | 0 | 0 | 0 | 1 | 0 | 0 | 0 | 0 | 2 | 0 | 0 | 0 | 0 | 1 | 0 | 0 | 0 | 0 |
| *Chimarra* (*Chimarra*) *emima* | 1 | 0 | 1 | 1 | 0 | 2 | 0 | 0 | 0 | 0 | 2 | 0 | 0 | 0 | 0 | 1 | 0 | 0 | 0 | 0 |
| *Chimarra* (*Otarrha*) *rossi* | 1 | 1 | 1 | 0 | 0 | 1 | 0 | 0 | 0 | 0 | 1 | 0 | 0 | 2 | 0 | 1 | 0 | 0 | 0 | 0 |

| Taxa | 21 | 22 | 23 | 24 | 25 | 26 | 27 | 28 | 29 | 30 | 31 | 32 | 33 | 34 | 35 | 36 | 37 | 38 | 39 | 40 | 41 |
| --- | --- | --- | --- | --- | --- | --- | --- | --- | --- | --- | --- | --- | --- | --- | --- | --- | --- | --- | --- | --- | --- |
| *C. akantha* | 1 | 1 | 0 | 0 | 0 | 1 | 1 | 0 | 1 | 0 | 0 | 2 | 2 | 1 | 1 | 2 | 0 | 0 | 0 | 0 | 0 |
| *C. anticheira* sp. n. | 0 | 0 | 0 | 0 | 0 | 0 | 1 | 0 | 1 | 0 | 0 | 2 | 2 | 1 | 1 | 2 | 1 | 1 | 0 | 0 | 0 |
| *C. camella* | 0 | 0 | 0 | 0 | 1 | 0 | 1 | 0 | 1 | 0 | 0 | 2 | 2 | 1 | 1 | 2 | 0 | 0 | 0 | 1 | 0 |
| *C. camura* | 0 | 0 | 0 | 0 | 0 | 0 | 1 | 0 | 1 | 0 | 0 | 2 | 2 | 1 | 1 | 2 | 0 | 1 | 0 | 1 | 0 |
| *C. chela* | 0 | 0 | 0 | 0 | 0 | 0 | 0 | 1 | 1 | 1 | 0 | 2 | 2 | 1 | 0 | 0 | 0 | 0 | 0 | 0 | 0 |
| *C. curvipenis* | 0 | 0 | 0 | 0 | 1 | 0 | 1 | 0 | 1 | 0 | 0 | 2 | 2 | 1 | 1 | 2 | 0 | 0 | 0 | 1 | 1 |
| *C. forcipata* | 0 | 0 | 0 | 0 | 0 | 0 | 0 | 1 | 1 | 1 | 0 | 2 | 2 | 1 | 0 | 0 | 0 | 0 | 0 | 0 | 0 |
| *C. heligma* | 0 | 0 | 0 | 0 | 0 | 0 | 1 | 0 | 1 | 0 | 0 | 2 | 2 | 1 | 1 | 1 | 0 | 0 | 0 | 1 | 1 |
| *C. heppneri* | 1 | 1 | 0 | 0 | 0 | 1 | 1 | 0 | 1 | 0 | 0 | 2 | 2 | 1 | 1 | 2 | 0 | 0 | 0 | 1 | 0 |
| *C.kontilos* | 0 | 1 | 0 | 0 | 0 | 0 | 1 | 0 | 1 | 0 | 0 | 2 | 2 | 1 | 1 | 2 | 0 | 0 | 0 | 1 | 1 |
| *C. latiforceps* | 0 | 0 | 0 | 0 | 0 | 0 | 1 | 0 | 1 | 0 | 0 | 2 | 2 | 1 | 1 | 2 | 1 | 1 | 1 | 0 | 0 |
| *C. majuscula* | 0 | 0 | 0 | 0 | 0 | 0 | 1 | 0 | 1 | 0 | 0 | 2 | 2 | 1 | 1 | 1 | 1 | 0 | 1 | 0 | 0 |
| *C. maldonadoi* | 0 | 0 | 0 | 0 | 0 | 0 | 0 | 1 | 0 | 0 | 1 | 1 | 1 | 0 | 0 | 0 | 0 | 0 | 0 | 0 | 0 |
| *C. merengue* | 0 | 0 | 0 | 0 | 0 | 0 | 0 | 1 | 0 | 0 | 1 | 1 | 1 | 0 | 0 | 0 | 0 | 0 | 0 | 0 | 0 |
| *C. mesodonta* sp. n. | 0 | 0 | 0 | 0 | 0 | 0 | 0 | 1 | 1 | 1 | 0 | 2 | 2 | 1 | 0 | 0 | 0 | 0 | 0 | 0 | 0 |
| *C. neblina* | 0 | 0 | 0 | 0 | 0 | 0 | 0 | 1 | 1 | 1 | 0 | 2 | 2 | 1 | 0 | 0 | 0 | 0 | 0 | 0 | 0 |
| *C. prolata* | 0 | 0 | 0 | 1 | 0 | 0 | ? | ? | ? | ? | ? | ? | ? | ? | ? | ? | 0 | 0 | 0 | 0 | 0 |
| *C. pusilla* | 0 | 0 | 0 | 0 | 0 | 0 | 0 | 1 | 1 | 1 | 0 | 2 | 2 | 1 | 0 | 0 | 0 | 0 | 0 | 0 | 0 |
| *C. rosalesi* | 0 | 0 | 0 | 1 | 0 | 0 | 0 | 1 | 1 | 1 | 0 | 2 | 2 | 1 | 0 | 0 | 0 | 0 | 0 | 0 | 0 |
| *C. simpliciforma* | 0 | 1 | 1 | 0 | 1 | 0 | 1 | 0 | 1 | 0 | 0 | 2 | 2 | 1 | 1 | 1 | 0 | 0 | 0 | 0 | 0 |
| *C. tortuosa* | 0 | 1 | 0 | 0 | 0 | 0 | 1 | 0 | 1 | 0 | 0 | 2 | 2 | 1 | 1 | 2 | 0 | 0 | 0 | 0 | 0 |
| *C. xingu* | 0 | 1 | 1 | 0 | 1 | 0 | 1 | 0 | 1 | 0 | 0 | 2 | 2 | 1 | 1 | 1 | 0 | 0 | 0 | 0 | 0 |
| *Chimarrhodella ulmeri* | 0 | 0 | 0 | 0 | 0 | 0 | 0 | 0 | 0 | 0 | 1 | 1 | 0 | 0 | 0 | 0 | 0 | 0 | 0 | 0 | 0 |
| *Chimarrhodella costaricensis* | 0 | 0 | 0 | 0 | 0 | 0 | 0 | 0 | 0 | 0 | 0 | 0 | 0 | 0 | 0 | 0 | 0 | 0 | 0 | 0 | 0 |
| *Chimarra* (*Curgia*) *banksi* | 0 | 0 | 0 | 0 | 0 | 0 | 0 | 1 | 0 | 0 | 0 | 0 | 0 | 0 | 0 | 0 | 0 | 0 | 0 | 0 | 0 |
| *Chimarra* (*Chimarra*) *emima* | 0 | 0 | 0 | 0 | 0 | 0 | 1 | 0 | 0 | 0 | 0 | 0 | 0 | 0 | 0 | 0 | 0 | 0 | 0 | 0 | 0 |
| *Chimarra* (*Otarrha*) *rossi* | 0 | 0 | 0 | 0 | 0 | 0 | 1 | 0 | 1 | 0 | 0 | 0 | 0 | 0 | 0 | 0 | 0 | 0 | 0 | 0 | 0 |
